# Age-related changes in P wave morphology in healthy subjects

**DOI:** 10.1186/1471-2261-7-22

**Published:** 2007-07-27

**Authors:** Rasmus Havmoller, Jonas Carlson, Fredrik Holmqvist, Alberto Herreros, Carl J Meurling, Bertil Olsson, Pyotr Platonov

**Affiliations:** 1 Department of Cardiology, Lund University Hospital, Lund, Sweden; 2 Department of Automatic Control, Valladolid University, Valladolid, Spain

## Abstract

**Background:**

We have previously documented significant differences in orthogonal P wave morphology between patients with and without paroxysmal atrial fibrillation (PAF). However, there exists little data concerning normal P wave morphology. This study was aimed at exploring orthogonal P wave morphology and its variations in healthy subjects.

**Methods:**

120 healthy volunteers were included, evenly distributed in decades from 20–80 years of age; 60 men (age 50+/-17) and 60 women (50+/-16). Six-minute long 12-lead ECG registrations were acquired and transformed into orthogonal leads. Using a previously described P wave triggered P wave signal averaging method we were able to compare similarities and differences in P wave morphologies.

**Results:**

Orthogonal P wave morphology in healthy individuals was predominately positive in Leads X and Y. In Lead Z, one third had negative morphology and two-thirds a biphasic one with a transition from negative to positive. The latter P wave morphology type was significantly more common after the age of 50 (P < 0.01). P wave duration (PWD) increased with age being slightly longer in subjects older than 50 (121+/-13 ms vs. 128+/-12 ms, P < 0.005). Minimal intraindividual variation of P wave morphology was observed.

**Conclusion:**

Changes of signal averaged orthogonal P wave morphology (biphasic signal in Lead Z), earlier reported in PAF patients, are common in healthy subjects and appear predominantly after the age of 50. Subtle age-related prolongation of PWD is unlikely to be sufficient as a sole explanation of this finding that is thought to represent interatrial conduction disturbances. To serve as future reference, P wave morphology parameters of the healthy subjects are provided.

## Background

Atrial fibrillation (AF) is the most common sustained arrhythmia of clinical importance. Over the last couple of decades, many important findings have contributed to the enhanced understanding of AF pathophysiology, but much still remains to be clarified. AF is normally characterized as permanent, persistent or paroxysmal (PAF), with the latter being more common in younger persons. Both variants might be asymptomatic but, unfortunately, PAF is none the less still associated with considerably increased mortality and morbidity, thus implying the need for early diagnosis and subsequent prophylactic measures [1].

Using band-pass filtered signal averaging technique, P wave duration (PWD) has been studied in PAF patients with inconsistent findings regarding possible clinical applications [2-7]. The morphology of the P wave has also been investigated [8,9]. Interatrial block (IAB), in the sense of delayed conduction between the right atrium (RA) and the left atrium (LA), has been associated with left atrial dysfunction and enlargement as well as atrial tachyarrhythmias including AF [10]. Bayés de Luna and co-workers provided the first ECG-criteria for IAB, including notes on P wave morphology [11]. In experimental settings, lesions of the muscular fibre band that abridges the anterior inter-atrial groove, known as Bachmann's bundle (BB), would reproduce this morphology [12] and suggests that BB would play a major part in the interatrial conduction. However, the use of standard 12-lead ECG is limited for the study of low voltage P waves and the need for high-quality signal acquisition has been suggested [13]. Using P wave triggered signal averaging of unfiltered P waves (PSAECG), our group, in a limited material, has previously observed significant differences in orthogonal P wave morphology between patients with PAF and control patients [14]. In that study, a suggested normal P wave morphology was observed in about half of the PAF patients. Although different properties of the P wave in healthy subjects have been investigated [15,16], their orthogonal P wave morphology has not yet been described in detail. Hence, in light of the above-mentioned changes of the P wave, that might represent a propensity for developing AF, the present study was aimed at exploring orthogonal-lead P wave morphology in healthy males and females and describing the normal P wave in detail.

## Methods

### Study population and ethics

The study population was recruited by advertising in local press. The subjects were interviewed and examined by a physician to ensure good health. A 12-lead ECG was performed and blood pressure was measured. Exclusion criteria included history of cardiovascular disease (coronary artery disease, hypertension, stroke, congestive heart failure, arrhythmia or valvular heart disease), excessive use of alcohol, or a pathological 12-lead ECG at rest, including non-sinus rhythm. A total of 120 healthy individuals were included. The study was approved by the Ethics Committee of Lund University (approval number LU 325–00). Written informed consent was obtained and the study complied with the Declaration of Helsinki.

### Data acquisition

A standard 12-lead ECG was recorded for 6 minutes with the subjects at rest. Data was acquired at a rate of 1 kHz and with a resolution of 0.625 μV. The data acquisition board used for the recordings was supplied by Siemens-Elema AB, Solna, Sweden.

### Signal processing and analysis

Specially designed software, developed in Matlab R14^® ^(The MathWorks, Inc., Natick, MA, USA), was used for signal processing and analysis. The method has been described in detail earlier [17,18]. In summary the following steps were taken in order to extract P wave data:

1. Orthogonal leads (X, Y and Z) were derived from the original 12-lead ECG using the inverse Dower transform [18,19].

2. High-pass filtering of the derived Leads X, Y and Z, to annul low frequency baseline drift (0.5 Hz cut-off) and 50 Hz bandstop filtering to minimize powerline interference was performed.

3. Automatic QRS-detection according to a previously described method [20].

4. Obtainment of data windows with 250 ms preceding the onset of each detected QRS-complex, assumed to include the P wave.

5. Time-shifting of data window to obtain maximum correlation in each Lead X, Y and Z. Merging of P waves with a cross-correlation coefficient > 0.90 in each individual lead.

6. Averaging of obtained P waves.

The onset and end of the resulting P waves were set manually. The averaged P waves were then classified manually regarding morphological type (see below).

### Definitions

**Figure 1:**
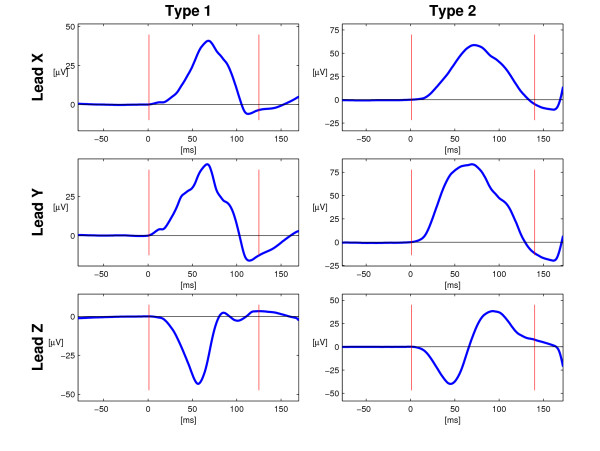
**P wave morphology Type 1 and 2**. The two principal orthogonal P wave morphology types earlier observed have been denoted Type 1 and 2. Type 1 is characterized by a positive Lead X, positive Lead Y, and negative Lead Z. Type 2 is characterized by a positive Lead X, a positive Lead Y, and a biphasic Lead Z with a transition from negative to positive. P wave onset and end are marked by vertical bars.

In this paper, the term "morphological type" is used to denote the P wave morphology in regard to all three orthogonal Leads X, Y, and Z. In our earlier report on P wave morphology two dominating and principally different types have been observed [14]: Firstly, a P wave morphology type with positive Lead X, positive Lead Y and negative Lead Z (denoted Type 1). Secondly, a P wave morphology type with positive Lead X, positive Lead Y, and biphasic Lead Z with a transition from negative to positive (Type 2). Type 1 and 2 are exemplified in Figure 1. A third type (Type 3), characterized by a biphasic Lead Y, corresponds to the advanced IAB described by Bayés de Luna [11] and also observed in patients with hypertrophic obstructive cardiomyopathy (HCM) in a recent study published by our group [21].

The onset and end of the P wave was defined as the earliest and latest activation in any Lead (X, Y or Z). P wave duration (PWD [ms]) was defined as the difference, in time, between the P wave onset and end.

**Figure 2:**
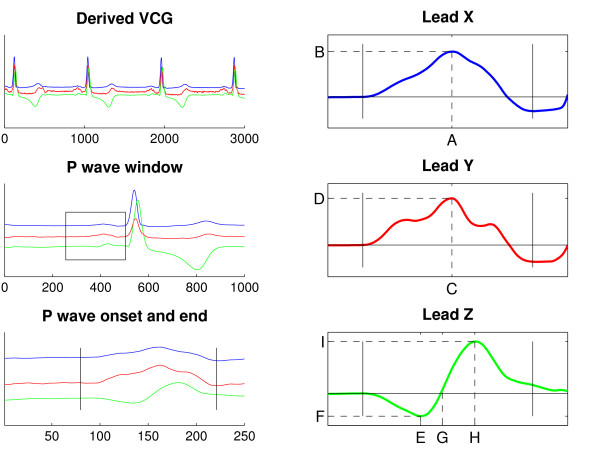
**P wave data acquisition and P wave parameters**. A schematic illustration of the method used for acquiring P wave data. After obtaining the derived leads, a QRS detection algorithm was applied. A data window of 250 ms preceding the QRS complex was created, supposedly containing the P wave. The window could be manually redefined if necessary. The data window was time-shifted and P waves then merged and averaged. P wave onset and end were set manually. The following P wave parameters were studied: location and amplitude of maximum in Lead X (X max pos, X max [A and B in the figure]), location and amplitude of maximum in Lead Y (Y max pos, Y max [C, D], location of zero crossing in Lead Z (Z zero pos [G]), and location and amplitude of minimum and maximum in Lead Z (Z min pos, Z min, Z max pos, Z max [E, F, H, I]. P wave onset and end are marked by vertical bars.

In Leads X and Y, the maximum amplitude was determined (X max, Y max) and also the distance from the onset of the P wave to the position of these maxima (X max pos, Y max pos). In Lead Z, the maximum and minimum amplitude was determined (Z max, Z min) along with their locations (Z max pos, Z min pos). In addition to this, the location of the zero crossing in Lead Z was measured when applicable (Z zero pos). The different parameters and a schematic illustration of the method used are shown in Figure 2.

### Statistics

All data is presented as mean ± standard deviation unless stated otherwise. Statistic evaluation was performed using the software StatView 4.5 (Abacus Concepts, Berkeley, CA, USA). The Mann-Whitney U-test and Kruskal-Wallis one-way analysis of variance were used for comparisons between populations of continuous data. For comparisons between groups of proportions the Chi-square test was used. A P-value less than 0.05 was considered statistically significant.

## Results

The study group was comprised by 60 men (age 50 ± 17 years) and 60 women (50 ± 16), (n.s.). The subjects were evenly distributed in groups of ten men and women for every decade from 20 years of age to 80. All subjects presented data, i.e. 6-minute registrations, available for analysis.

The males were significantly taller and heavier than the females (179 ± 7 cm vs. 165 ± 6 cm, P < 0.01 and 78 ± 9 kg vs. 65 ± 8 kg, P < 0.01). They also had lower heart rate (68 ± 9 bpm vs. 73 ± 10 bpm, P < 0.01) and higher systolic and diastolic blood pressure (128 ± 13 mmHg vs. 122 ± 13 mmHg, P < 0.01 and 78 ± 7 mmHg vs. 74 ± 7 mmHg, P < 0.01 respectively). With increasing age, a slight increase of systolic and diastolic blood pressure was also observed but still within normal limits.

P wave duration (PWD) was longer in males (127 ± 11 ms vs. 121 ± 12 ms, P < 0.01). PWD also increased significantly with increasing age (P < 0.05). When comparing PWD between subjects aged below and above 50, a slight but significant increase was observed in the older group (121 ± 13 ms vs. 128 ± 12 ms, P < 0.005). Baseline data is presented in Table 1.

**Table 1:** Baseline data of the study population

	**Height**	**Weight**	**BMI**	**BP syst**	**BP diast**	**HR**
**All**	172 ± 10	71 ± 11	24 ± 2	124 ± 12	76 ± 7	71 ± 10
**Men**	179 ± 7	78 ± 9	24 ± 2	128 ± 13	78 ± 7	68 ± 9
**Women**	165 ± 6	65 ± 8	24 ± 2	122 ± 13	74 ± 7	73 ± 10
**P-value**	**< 0,001**	**< 0,001**	**0,28**	**0,01**	**0,01**	**< 0,001**
**20–29 yrs**	165 ± 8	58 ± 7	21 ± 1	110 ± 6	65 ± 4	73 ± 9
**30–39 yrs**	167 ± 6	63 ± 7	22 ± 2	121 ± 8	74 ± 4	67 ± 8
**40–49 yrs**	162 ± 5	64 ± 3	24 ± 1	115 ± 7	75 ± 4	75 ± 9
**50–59 yrs**	163 ± 4	67 ± 5	25 ± 2	123 ± 11	82 ± 6	73 ± 9
**60–69 yrs**	165 ± 5	68 ± 8	25 ± 2	132 ± 13	77 ± 4	76 ± 7
**70 < yrs**	165 ± 7	69 ± 11	25 ± 3	127 ± 19	72 ± 5	77 ± 15
**P-value**	**0,66**	**0,60**	**< 0,001**	**< 0,001**	**< 0,001**	**0,28**

The study population baseline data is illustrated above as follows: height [cm], weight [kg], BMI (body mass index) [kg/m^2^], BP syst (systolic blood pressure) [mmHg], BP diast (diastolic blood pressure) [mmHg], and HR (heart rate) [beats per minute].

With the exception of higher P wave amplitude in Lead Y (Y max) for females compared to males, the only significant gender or age-differences regarding amplitudes or positions of P wave maxima or minima were seen in Lead Z. Here females had lower amplitudes of maxima and also earlier location of minima. With older age, the amplitude of the maxima in Lead Z increased while the zero-crossing was located earlier thus making the positive phase of the P wave in Lead Z greater. The numerical data and P-values of PWD as well as the amplitudes and temporal markers of X max, Y max, Z max, Z min and Z zero are shown in Table 2.

**Table 2:** P wave parameter and morphology data

	**P wave duration**	**X max position**	**X max amplitude**	**Y max position**	**Y max amplitude**	
**All**	123 ± 12	65 ± 10	51 ± 15	60 ± 10	80 ± 30	
**Men**	127 ± 11	67 ± 10	51 ± 10	60 ± 8	74 ± 21	
**Women**	121 ± 12	64 ± 10	52 ± 18	59 ± 10	87 ± 35	
**P-value**	**0,004**	**0,19**	**0,85**	**0,57**	**0,05**	
**20–29 yrs**	117 ± 12	63 ± 10	40 ± 9	53 ± 8	59 ± 27	
**30–39 yrs**	117 ± 11	63 ± 10	50 ± 12	57 ± 11	85 ± 29	
**40–49 yrs**	121 ± 13	64 ± 9	52 ± 12	61 ± 8	95 ± 26	
**50–59 yrs**	120 ± 13	69 ± 7	68 ± 22	66 ± 9	114 ± 50	
**60–69 yrs**	123 ± 15	62 ± 16	51 ± 23	59 ± 12	92 ± 29	
**70 < yrs **	125 ± 9	64 ± 6	53 ± 15	59 ± 11	77 ± 18	
**P-value**	**0,02**	**0,57**	**0,07**	**0,21**	**0,37**	

	**Z min position**	**Z min amplitude**	**Z zero position**	**Z max position**	**Z max amplitude**	**P wave morphology**

**All**	41 ± 7	-30 ± 10	65 ± 13	86 ± 15	20 ± 10	45/73/2
**Men**	43 ± 7	-32 ± 11	65 ± 12	89 ± 15	25 ± 10	18/42/0
**Women**	39 ± 6	-29 ± 9	64 ± 13	85 ± 15	19 ± 11	27/31/2
**P-value**	**0,004**	**0,09**	**0,26**	**0,10**	**0,003**	**0,001**
**20–29 yrs**	42 ± 5	-26 ± 7	72 ± 11	87 ± 17	9 ± 5	12/8/0
**30–39 yrs**	40 ± 4	-31 ± 10	64 ± 10	85 ± 16	16 ± 11	10/10/0
**40–49 yrs**	38 ± 8	-27 ± 10	67 ± 22	82 ± 22	18 ± 11	12/8/0
**50–59 yrs**	38 ± 6	-29 ± 11	57 ± 8	79 ± 6	22 ± 10	1/18/1
**60–69 yrs**	36 ± 5	-29 ± 6	60 ± 9	92 ± 15	21 ± 9	5/14/1
**70 < yrs**	41 ± 6	-33 ± 9	63 ± 13	84 ± 6	26 ± 12	2/18/0
**P-value**	**0,18**	**0,59**	**0,02**	**0,35**	**< 0,001**	**< 0,001**

P wave parameter data is provided above. All positions and P wave duration (PWD) are expressed as [ms] and all amplitudes as [μV]. In the column "P wave morphology", the distribution of different P wave morphology types is provided (Type 1/Type 2/Atypical).

### P wave morphology

Of the 120 subjects, 118 had P wave morphology types consistent with those observed in earlier reports; 45 Type 1 (37%, 18 males, 27 females), and 73 Type 2 (61%, 42 males, 31 females). Two subjects (both females) had P wave morphologies that were classified as atypical, i.e. neither Type 1, 2 or 3.

**Figure 3:**
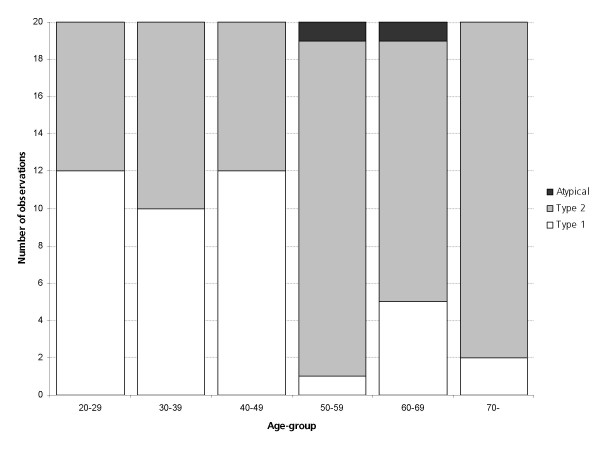
**Distribution of P wave Type 1 and 2 in different age groups**. The study population was evenly distributed in decades. In the diagram, the distribution of P wave morphology types is shown for different age groups. P waves of Type 1 (white) are more common in the younger population and those of Type 2 (shaded) are significantly more common in the older age groups. Two cases of atypical P wave morphologies (dark grey) were also observed.

When comparing the distribution of P wave morphology types in the different age groups it was found that the Type 2 morphology was more common with increasing age (P < 0.01). There was also a significant (P < 0.001) increase in Type 2 morphology in subjects older than 50 when comparing equally large groups of subjects younger and older than 50. In the younger group 26 of 60 cases of Type 2 morphology were observed in comparison to 50 of 60 in the older group. The distributions of P wave morphology types in different age groups are illustrated in Figure 3.

### Dynamic changes of P wave morphology

**Figure 4:**
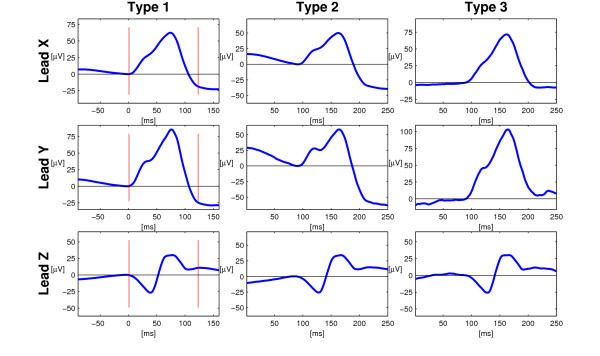
**Example of a registration with many P wave groups**. In 8 % of the cases, more than one P wave group was observed. However, in no case did the plurality seem to reflect true differences of P wave morphology. An example of this is shown above where the three P wave groups all have the same gross P wave morphology, Type 2.

In 92 % of the cases only one group of P wave morphologies was observed during 6-min long recording. In the remaining cases (8%), additional groups of P waves with a cross-correlation coefficient < 0.90 were obtained. The P waves from different groups appeared to be very similar in shape and of the same morphological type (Figure 4).

## Discussion

### Main findings

In the present study, orthogonal ECG registrations from 120 healthy adults of varying age were analysed using PSAECG. Two main P wave morphologies were observed (Type 1 and 2). Type 2 morphology, associated with PAF and other arrhythmia-prone patients, was more common in healthy subjects than earlier perceived [14] and highly dominant after the age of 50. Intraindividual variation was minimal.

### Interatrial conduction

In a report on the anatomy of the human atria Ho and co-workers denoted BB, the muscular fibre band that abridges the anterior inter-atrial groove, the "superhighway for inter-atrial conduction" [22]. However, the presence of additional smaller (and possibly more vulnerable) inter-atrial muscular connections crossing superior, inferior, and posterior parts of the inter-atrial groove as well as connections between the wall of the coronary sinus (CS) and the LA or RA was noted. This morphological variation was confirmed in post-mortem pathohistological examinations of human hearts [23].

From an electrophysiological standpoint, BB has been considered the preferential conductive route from RA to LA but other transseptal pathways, close to Koch's triangle and also in the CS area have been recognized [24]. The functional aspects of interatrial connections have, during the last decade, been investigated by several groups [25-29]. The findings have not been uniform but in short, right-to left atrial conduction rarely occurs via the septum, but rather via multiple inter-atrial conduction routes. Anterior transseptal breakthrough corresponding to BB was observed. In addition to this anterior-inferiorly located as well as posterior inter-atrial connections were reported and the latter are in some reports considered equally as important as BB [25-29]. It is however worth noticing that most of the studies were performed in patients with PAF, thus the conductive findings might not necessarily apply to sinus rhythm of healthy subjects.

### The genesis of the P wave and age-related changes of P wave morphology

PSAECG has previously been used by our group to study orthogonal P wave morphology in patients with PAF, HCM and control patients [14,21]. Three types of P wave morphology have been observed (as described in Methods) and the findings of the present study are in consistency with these findings. Type 1 is thought to represent an activation sequence directed right-left, superior-inferior and posterior-anterior. This is best explained by activation via posteriorly located interatrial conduction routes. Type 2 with biphasic Lead Z consequently differs in regards to an anterior-posterior-anterior activation sequence, best explained by interatrial conduction via the anteriorly located BB. The assumption that the changes in the terminal part of Lead Z would represent LA activation is supported by data recently published by Lemery et al [30]. Type 3, earlier reported in HCM patients, was logically not observed in the healthy population of the present study.

In the reported material, Type 2 morphology is significantly more common with increasing age with a marked increase in prevalence after the age of 50. We did not observe any unexpected changes of the characteristics of the study group that would explain these findings that implicate altered RA-LA activation. For instance, only slight prolongation of mean PWD (ΔPWD = 7 ms) with age was observed and the longer PWD in men could possibly be explained by their heavier stature. As explained above, interatrial conduction can occur via several pathways. One could speculate that age-related changes in the human heart such as fibrosis that have been reported and suggested to play a role in atrial reentrant mechanisms [31] could affect the interatrial muscular bundles thus altering interatrial conduction. Possibly the most crude structure, i.e. BB, would be the least vulnerable and the most likely to remain functional with advancing age. This fits well with our findings of Type 2 morphology being more common in the older age groups. Furthermore, the Type 2 morphology has earlier been reported as common in PAF patients, which is interesting considering the well-known correlation between age and incidence of AF. This morphology type is similar to the one observed in 61% of the healthy subjects of the present study. This indicates that this type of inter-atrial conduction is more common in healthy individuals than earlier perceived.

However, other causes of altered P wave morphology are also plausible. These include variations in the atrial pacemaker complex and sinus node morphology but also differences of conduction velocities [32]. Furthermore it is well known that changes in autonomous discharge as well as heart rate variability affect electrophysiological properties such as PWD [33-36].

However, intraindividual variation of P wave morphology during 6-min long recordings was minimal in the present study. This could indicate that interatrial conduction propagation on an individual basis is stable and that the changes that occur are stable as well. These age-related changes in P wave morphology are therefore unlikely to be caused solely by the age-related decrease in conduction velocity reflected in marginal prolongation of PWD, but could best be explained by changes in the function of interatrial conductive pathways.

### Limitations of the study

Even though the selection process was aimed at including only healthy asymptomatic subjects with normal blood pressure, no known diseases and not taking any medication it cannot be ruled out that some subjects with asymptomatic AF may have been included. However, in our opinion possible impact of these single individuals on the overall results would be negligible. Furthermore, influence of atrial dimensions on P wave morphology that has been demonstrated earlier cannot be excluded since echocardiography data for the study group was not available.

## Conclusion

The presented study describes orthogonal P wave morphology of a material comprised of healthy men and women and provides a reference material for future research. Changes of signal averaged orthogonal P wave morphology (biphasic signal in Lead Z), earlier reported in PAF patients, are common in healthy subjects and appear predominantly after the age of 50. Subtle age-related prolongation of PWD is unlikely to be sufficient as a sole explanation of this finding that is thought to represent interatrial conduction disturbances. However, further studies are needed to determine the electrophysiological explanation for these changes and its possible role as a substrate for arrhythmia.

## Competing interests

The author(s) declare that they have no competing interests.

## Authors' contributions

RH participated in the processes of study design, data acquisition and analysis. JC, AH and PP contributed to study design, data acquisition and analysis. CM and BO took part in study design and analysis. All authors participated in the process of drafting the manuscript as well as read and approved the final manuscript.

## Pre-publication history

The pre-publication history for this paper can be accessed here:


